# Appraisal of the Pre-Emptive Effect of Lactoferrin Against Chromium-Induced Testicular Toxicity in Male Rats

**DOI:** 10.1007/s12011-023-03605-3

**Published:** 2023-03-06

**Authors:** Sahar S. Abd El-Rahman, Nadia M. Ashwish, Merhan E. Ali

**Affiliations:** 1 Department of Pathology, Faculty of Veterinary Medicine, Cairo University, Giza, 12211 Egypt; 2 Department of Cell and Molecular Biology-PhD, 2018, College of Veterinary Medicine and Biomedical Sciences, Colorado State University, Fort Collins, CO USA

**Keywords:** Chromium, Lactoferrin, Testes, Oxidative stress, Pro-inflammatory cytokines, Apoptosis

## Abstract

Lactoferrin (LCF), a potent naturally occurring antioxidant, is a crucial component in preventing potassium dichromate (PDC) toxicity. The goal of the current work was to study the potential efficacy of LCF in preventing PDC(CrVI)-induced testicular toxicity and oxidative injury in rats. Six groups of male rats of Wistar stain were randomly categorized into: group 1, which served as the control; group 2 and 3 received LCF (200 and 300 mg/kg orally, respectively); group 4 received PDC (2 mg/kg i.p.); group 5 and 6 pretreated with LCF, followed by PDC as in group 4 with 90 min apart for 28 days. PDC-intoxicated rats showed a significantly altered spermogram with abnormal sperm morphology. PDC significantly upregulated serum FSH and downregulated testosterone levels. Additionally, PDC decreased the levels of testicular key antioxidant biomarkers (catalase (CAT), superoxide dismutase (SOD), and glutathione (GSH)) with elevated lipid peroxidation marker (TBARS) and testicular chromium content. Moreover, it upregulated testicular proinflammatory cytokines, IL-1, IL-6, IL-10, and TNF-α, induced histopathological changes in testes with significant immunohistochemical expression of FasL and moderate expression of Nrf2. Pretreatment with LCF significantly mitigated PDC-induced testicular toxicity by enhancing spermogram, improving hormonal levels, restoring testicular oxidant/antioxidant balance, and decreasing testicular IL-1, IL6, IL-10, and TNFα levels, and amending both FasL and Nrf2 immunohistochemical-expression. Additionally, LCF improved testicular histopathological picture and spermatogenesis. Our results highlight the importance of LCF as a superior protective modulator of PDC-induced testicular injury.

## Introduction

The prevalent use of metals on a global scale has increased concern about ecological contamination, which has stimulated research in toxicological studies. Recent increases in heavy metal toxicity can be attributed to domestic, agricultural, and industrial activities that pose a grave environmental threat [1]. PDC is one of the heavy metals used extensively in industries including chrome plating, leather tanning, pigment production, welding, stainless steel, and cement production. Chromium is also utilized in the glass, ceramic, and photographic industries. It is also utilized as an anti-corrosion agent, catalytic converter output for automobiles, in cooling plants, and for a variety of other applications [2]. Unregulated applications can pollute water and soil with chromium, as their discharge contains chromium. Because chromium incorporates in the food chain via plants, it poses a significant threat to human health. There are two oxidation states of chromium, hexavalent (Cr-VI), and trivalent (Cr-III). It was found that Cr-VI is one hundred to one thousand times more toxic than the trivalent state [3]. Chromium uses nonspecific anion transporters1 to cross the cellular membranes. After entering the cell, the hexavalent chromium is undergone reduction to generate reactive intermediates, including Cr-V, Cr-IV, and Cr-III, as well as reactive oxygen species (ROS) [4]. They can induce base modifications, DNA strand fragmentation, and lipid peroxidation, impairing the integrity of cells as well as promoting both toxic and mutagenic impacts [5]. Furthermore, hexavalent chromium causes many chronic and acute toxicity problems such as genotoxicity, carcinogenicity, immunotoxicity, neurotoxicity, and dermatotoxicity to animals and human exposure to this metal, along with environmental hazards [6]. Hexavalent chromium can cause severe reproductive abnormalities, including an increased risk of sperm abnormalities and poor-quality sperm, resulting in infertility or increasing the risk of developmental issues in children [7].

Several animal species, such as rats [8], mice [9], and bonnet monkeys [10], have exhibited chromium-induced sperm abnormalities and decreased sperm count. The male reproductive system is extremely susceptible to several environmental agents, such as toxicants, pollution, medications, and radiation. Consequently, the population of industrialized nations is threatened by decreased rates of fertility [11].

As a preventative measure, the global trend is toward utilizing natural substances as therapeutic antioxidants. Due to their low cost, natural antioxidants are viewed as a potential substitute for synthetic antioxidants, as they have no harmful effects on the human body and are highly convenient with dietary intake [12]. LCF is a naturally occurring iron-binding glycoprotein found in the majority of mammalian exocrine secretions like intestinal and bronchial (mucosal) secretions, milk, saliva, and tears [13]. Additionally, it is found in the neutrophil’s secondary granules. LCF has several pharmacological properties, which are interceded via particular receptors on many cellular surfaces [14]. Among these properties are antimicrobial, antiviral, antitumor [15], anti-parasitic [16], anti-inflammatory [17], and antioxidant [18]activities, as well as potent immunoregulatory properties [15, 19].

As far as we are aware, this research is the first to investigate the potential protective role of LCF to prevent rats’ testicular injury from PDC toxicity.

## Materials and Methods

### Chemicals

PDC was purchased from Sigma Aldrich (207802), USA, and LCF from Sigma, Aldrich (L9507), USA. It was supplied as a white powder and was freshly prepared by dissolving it in water for oral administration.

### Ethical Statement

The Institutional Animal Care and Use Committee at Cairo University approved all protocols for the experimental design and handling of animals used in this study (CUIIF2622), with precautions taken to alleviate the animal’s suffering.

### Animals and Treatment

**Fig. 1:**
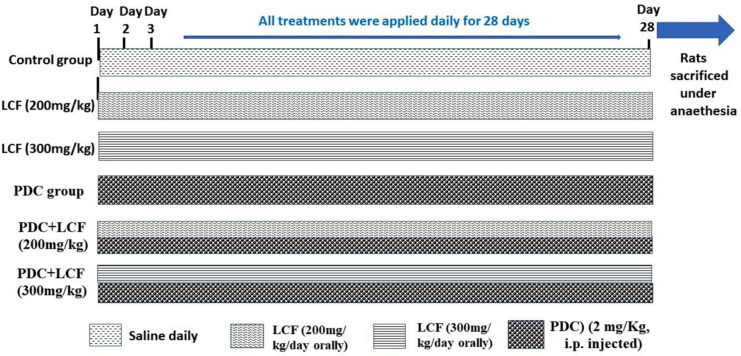
Schematic representation of the experimental design

Sixty male Wistar rats (200–220 g) were secured from the National Research Center’s animal house in Dokki, Cairo, Egypt. Rats were kept in stainless-steel cages and offered commercial rat pellets containing 44% soybean powder, 10% wheat bran, 22% net protein, 3.3% fibers, 4.7% fats, methionine, fish meal, salts (calcium phosphate, sodium chloride, and calcium carbonate), and molasses (Cairo Agriculture Development Company, 6th October city, Egypt). Water and food were available ad libitum. Animals were given time to get used to the lab environment (temperature, 21 °C; photoperiod, 7 AM to 7 PM) 1 week before the start of the experiment. They were divided into six groups: the first group, which served as a standard control group, got saline; groups 2 and 3; rats received LCF at doses of 200 and 300 mg/kg/day orally by gavage) [20, 21], respectively, for 28 days. In group 4, rats received daily PDC (2 mg/kg) [22], which was freshly prepared by dissolving the powder in saline solution and intraperitoneally administrated at a dose of 2 mg/kg. In groups 5 and 6, rats were concomitantly administrated LCF and PDC, starting with LCF in groups 2 and 3, respectively, followed by PDC administration in group 4 with 90 min intervals for 4 weeks (Fig. 1).

### Sample Collection

At the conclusion of the experiment, rats were fasted overnight, their final body weight was recorded, and then they were anesthetized with intravenous ketamine (75 mg/kg, i.p.). In a clean tube, blood samples were compiled from the eye’s inner canthus for serum separation, followed by cervical decapitation of all animals. The collected blood samples were subjected to centrifugation for serum separation. The obtained serum samples were then stored for further examination at − 20 °C.

After decapitation, sperm sample collection from the epididymis tail was done.

The testes of both sides were dissected and weighed. Each animal’s right testis was fixed in Bouin’s solution for histopathological analysis, whereas the left testis was frozen at − 20 °C for further homogenization and biochemical analysis.

### Determination of Epididymal Sperm Head Counts, Sperm Motility, Abnormalities, and Viability

In physiological saline (5 ml), the epididymis was minced using anatomical scissors to collect the sperm samples before being incubated for 2 min at 32 °C. The sperm count and mass motility were evaluated following methods adopted by Linder et al. [23]. On a Neubauer hemocytometer, an aliquot of the previous solution was placed, and counting the motile sperms was carried out at 400 × magnification microscopic power in at least three randomly selected fields for each sample. First, we counted the non-motile sperm, followed by the total sperm number.

The ratio of the number of motile sperm to all the sperm counted was used to calculate the sperm motility percent. The percentage of various sperm abnormalities was evaluated [24]. Slides with spermatozoa smears were prepared and stained with Wells and Awa stain to detect morphological abnormalities. Other smears were stained with 1% eosin B and 5% nigrosine to distinguish between living and dead sperms. About 100 sperm cells were counted on each slide at 100 × magnification light microscopic field.

### Hormonal Analysis

Serum testosterone level was determined utilizing a competitive-ELISA kit (Leader Trade Co., Giza, Egypt) according to the method described by Demetrius [25]. While the serum level of FSH was determined using sandwich-ELISA kits (Leader Trade Co., Egypt) according to the method mentioned by Beastall [26].

### Preparation of Testicular Tissue Homogenate

Each animal’s testicular specimen was homogenized (10% w/v) in 0.01 mol/L of ice-cold sodium–potassium phosphate and 1.15% KCl buffer (pH 7.4), followed by 20 min of centrifugation at 10 000 g (4 °C) for 20 min. The obtained supernatant was utilized for further determination of the following parameters.

### Chromium Residue Determination in Testicular Tissue

Chromium residue was estimated in testicular tissue utilizing atomic absorption spectrophotometer (AAS, unicam 969) [27]. Testicular tissue samples were wet digested in a solution of hydrogen peroxide and nitric acid (1:3). Then the samples were kept aside for 10 min before being heated on a hot plate for 1 h at 60 to 70 °C with the addition of nitric acid until becoming colorless.

### Determination of TBARS, Glutathione Content, and Enzymatic Antioxidant Levels in Testes

SOD activity [28], reduced GSH content [29], and catalase (CAT) activity [30] were all determined in the prepared testicular tissue homogenate of each animal in each group.using specific diagnostic kits (Biodiagnostic Co., Egypt, CAT. NO.; SD2521, GR2511, and CA2517, respectively). Thiobarbituric acid-reactive substances (TBARS) were also assessed in the same homogenates using a diagnostic kit (ELK Biotechnology CO. Ltd., Wuhan, China) (ELK8905) [31].

### Determination of Proinflammatory Cytokines Levels

Testicular contents of proinflammatory cytokines encompassing interleukins (IL-1, IL-6, and IL-10) and TNF-α were assessed in the prepared homogenate according to the manufacturing instructions using ELISA commercial kits (Cusabio Biotech®).

### Histopathological Evaluation

Testicular specimens previously fixed in Bouin’s solution were processed, embedded in paraffin, serially sectioned at 4–5-μm thickness, then stained with Hematoxylin and Eosin (H&E) [32] for light microscopic evaluation. On the basis of a modified Holstein’s system, a semiquantitative analysis of histopathological testicular lesions was performed (Table 1). At least ten randomly selected fields from each animal slide were used to score the observed histopathological lesions, followed by a cumulative figure for each group.

**Table 1:** Testicular samples’ histopathological scoring according to modified Holstein’s system

Score	Modified Holstein’s scoring system
10	Complete spermatogenesis with several spermatozoa as well as spermiation zones
9	Slightly diminished spermatogenesis; lower spermatozoa number, a few zones of spermiation
8	Distinguished reduction in spermatogenesis; a small number of spermatozoa without spermiation
7	Spermatogenesis is decreased; absence of spermatozoa, only spermatids without spermiation
6	Severe decrease in spermatogenesis; with fewer spermatids and decreased the germinal epithelium height
5	Spermatogenesis arrest at the primary spermatocyte stage; the seminiferous tubule's lumen is bordered with many spermatocytes
4	Spermatogenesis arrests at the primary spermatocytes stage with the presence of a few primary spermatocytes
3	Stop at the spermatogonia stage; a type of spermatogonia multiplies but does not develop into mature cells of spermatogenesis
2	There are Sertoli cells only but no germ cells
1	Absence of germ cells and Sertoli cells. Connective tissue ground substance replaces the seminiferous tubule

Histomorphometric analysis of various parameters of the seminiferous tubules was carried out, including area, circumference, and diameter in randomly selected ten seminiferous tubules utilizing image analysis software (ImageJ, 1.46a, NIH, USA)

### Immunohistochemical Examination

Immunohistochemical analysis of FasL and Nrf2 expression in the testicular tissue of each group was conducted. The paraffin-embedded testicular tissues were sectioned into 5 μm on slides (positively charged) to detect the immune expression of FasL and Nrf2. Sections were dewaxed, rehydrated, and exposed to antigen retrieval by autoclaving for 10 min at 120 °C in 10 mM citrate buffer (pH 6). Blocking of endogenous peroxidases was done using 0.3% H2O2 in methanol for 15 min. After washing the slides in PBS, a blocking buffer was added before incubation for 30 min at an ambient temperature. In addition, primary polyclonal ad monoclonal antibodies for Nrf2 and FasL were added at dilutions of 1:200 and 1:100 in PBS and incubated for 30 min. The secondary antibody of biotinylated polyvalent type (Cat No. 32230, Thermo Scientific Co., U.K.) was added to tissue sections before coincubation for 30 min. Visualization of the reaction was done by adding DAB Substrate Working Solution to the tissue, and hematoxylin stain was utilized as a counterstain [33]. Seven high-power microscopic fields were used to quantify the area % of the positive brown areas of each marker's expression using image analysis software (Image J, 1.46a, NIH, USA).

### Statistical Analysis

The obtained data were statistically analyzed using the SPSS 25.0 software, and data were displayed as means ± SE. ANOVA (one-way analysis of variance), followed by the Tukey post-hoc test, was used to clarify comparisons between several groups. Values were deemed statistically significant at *P* < 0.05. Nonparametric analysis frequency data were compared using the Kruskal–Wallis H test and the Mann–Whitney U test. The nonparametric data were expressed as a median. The difference was deemed significant at *P* < 0.05.

## Results

### Lactoferrin Maintained Body and Testicular Weights in PDC-Intoxicated Rats

**Fig. 2:**
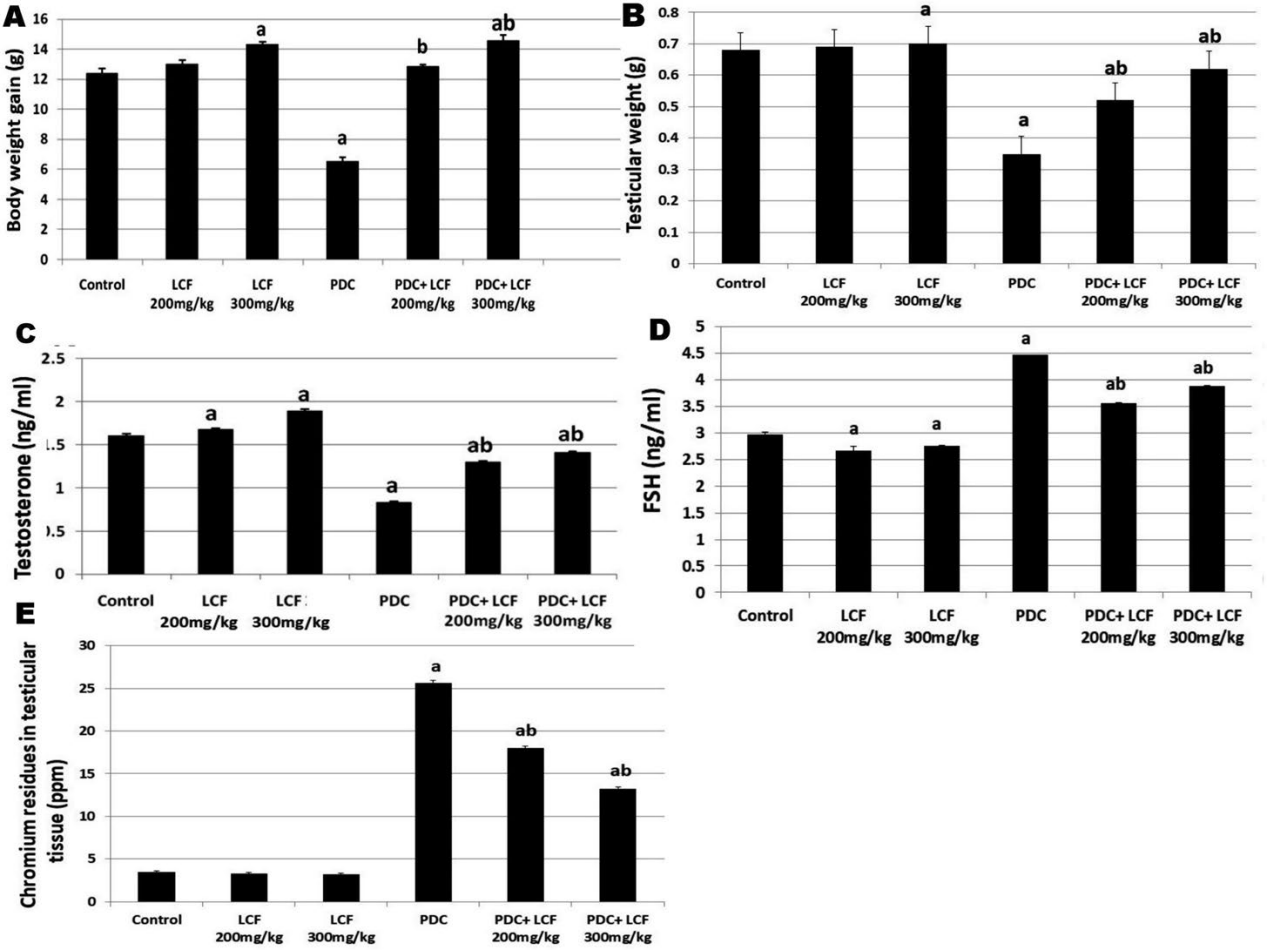
Effect of Lactoferrin pretreatment on body (**a**) and testicular (**b**) weights, serum testosterone (**c**) and FSH (**d**) levels as well as chromium residue in testicular tissue  in PDC administrated rats. Values are presented as mean ± SE. a refers to statistically significant difference from the control group at *P* < 0.05. b refers to statistically significant difference from PDC administrated group at *P* < 0.05

Administration of PDC resulted in a marked decline (*P* < 0.05) in the body as well as testicular weights compared to controls. However, pretreatment with LCF (200 and 300 mg/kg) significantly restored both testicular and body weight compared with the PDC group (Fig. 2A and B). Compared with controls, no marked changes were determined in the LCF alone (200 mg) treated group. However, LCF alone (300 mg) showed a substantial elevation in both body and testicular weights compared with the control.

### Lactoferrin Curtailed PDC-Induced Alterations in Sperm Head Counts, Motility, Abnormalities, and Viability in PDC-Intoxicated Rats

A substantial decline (*P* < 0.05) in sperm head counts, motility, as well as viability, was discerned in the epididymis of PDC-treated rats as compared to that of the control and LCF-administered groups (Table 2). LCF administration at 200 and 300 mg/kg doses significantly increased the last sperm parameters. In contrast, treatment of rats with both doses of LCF before PDC administration significantly reversed (*P* < 0.05) the PDC-induced decrease in sperm counts, motility, and viability. In addition, PDC administration led to a marked elevation in sperm morphological abnormalities compared to controls and the LCF-treated group alone. By comparing the LCF-treated group to the PDC-administered group, sperm morphological abnormalities were significantly reduced in the LCF-treated group.

**Table 2:** Effect of pretreatment of LCF on sperm head count (× 10^5^/ml), sperm motility (%), and percent of dead and abnormal sperms in PDC administrated rats

Groups items	Control	LCF 200 mg/kg	LCF 300 mg/kg	PDC	PDC + LCF 200 mg/kg	PDC + LCF 300 mg/kg
Sperm head count(× 10^5^/ml)	165.14 ± 2.68	180 ± 1.25^a^	189 ± 1.05^a^	92 ± 1.94^a^	124 ± 1.81^ab^	140.86 ± 1.35^ab^
Sperm motility (%)	76.43 ± 0.78	80.57 ± 1.2^a^	89.57 ± 0.92^a^	47 ± 0.81^a^	55.86 ± 0.63^ab^	69 ± 0.95^ab^
Dead sperm (%)	25.43 ± 0.65	20.23 ± 0.61^a^	19.14 ± 0.67^a^	56.86 ± 0.91^a^	45.14 ± 0.83^ab^	33.71 ± 0.75^ab^
Abnormal sperm (%)	13 ± 0.44	11.14 ± 0.34^a^	8.86 ± 0.34^a^	24.43 ± 0.53^a^	20 ± 0.53^ab^	16.57 ± 0.48^ab^

Values are presented as mean ± SE. ^a^ refers to a statistically significant difference from the control group at *P* < 0.05. ^b^ refers to a statistically significant difference from PDC administrated group at *P* < 0.05

### LCF Rectified PDC-Induced Changes in Hormone Levels

Serum testosterone levels were substantially diminished (*P* < 0.05) in rats administrated PDC, while FSH was substantially elevated (*P* < 0.05), as compared with the control group and LCF control groups (Fig. 2C and D). Groups pretreated with LCF at doses of 200 and 300 mg/kg before PDC administration showed significant dose-related attenuation in the testosterone levels and FSH altered by PDC compared to the PDC group.

### LCF Decreased Chromium Residues in the Testicular Tissue of PDC-Intoxicated Rats

The assay of the atomic absorption spectrophotometer of Cr residues in testicular tissue in all groups displayed marked (*P* < 0.05) elevation in chromium concentration in PDC-intoxicated rats compared to control and LCF administrated groups. However, the pretreatment with LCF (200 and 300 mg/kg) significantly (*P* < 0.05) lowered chromium concentration in a dose-related reaction compared with that of the PDC group (Fig. 2E).

### LCF Suppressed Oxidative Stress Markers’ Levels in the PDC-Intoxicated Rats

**Fig. 3:**
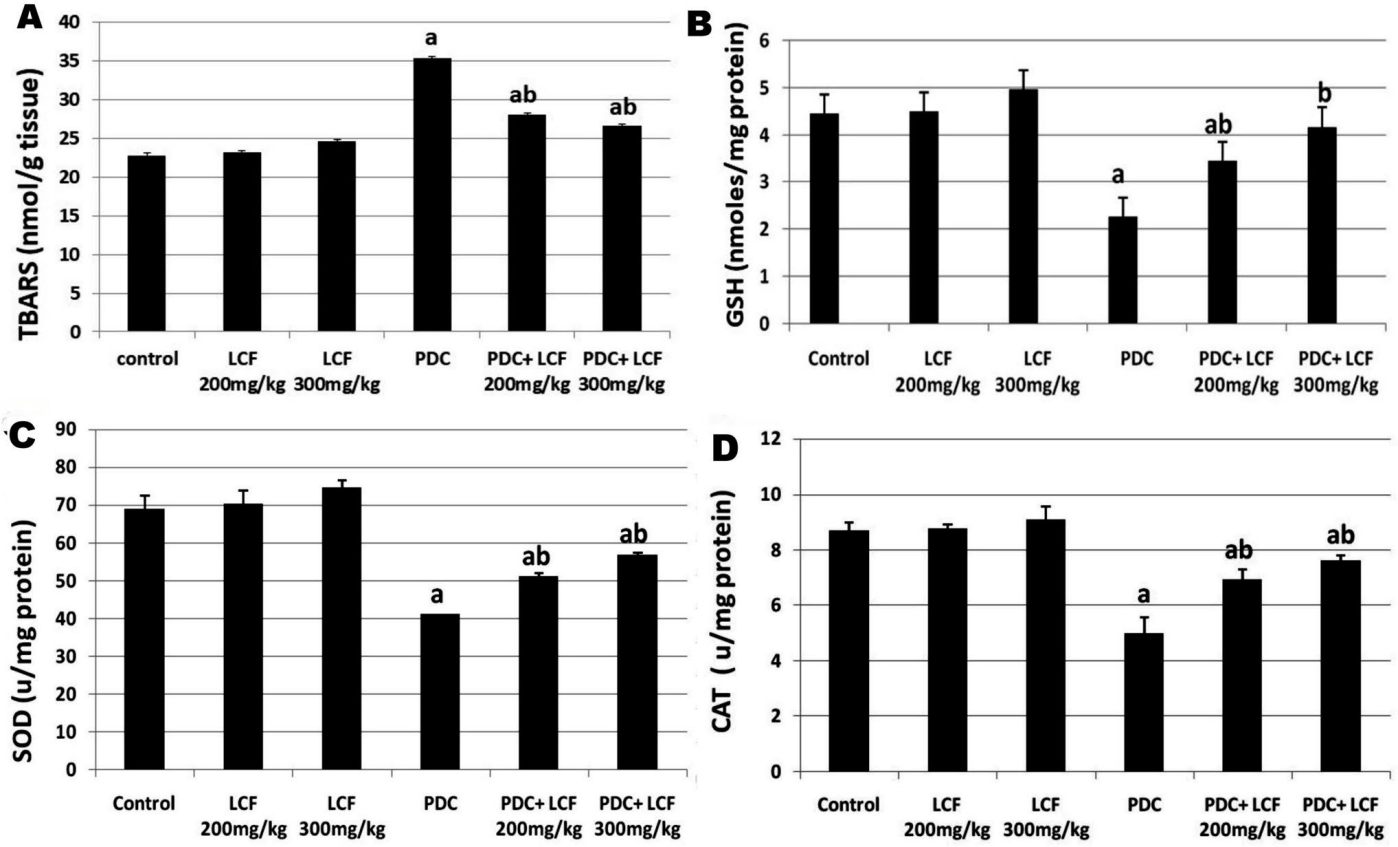
Effect of Lactoferrin on oxidative stress markers; TBARS (**a**), GSH content (**b**), SOD (**c**), and CAT activities in testicular tissue homogenate of PDC rats. Values are presented as mean ± SE. ^a^ refers to statistically significant difference from the control group at *P* < 0.05. ^b^ refers to statistically significant difference from PDC administrated group at *P* < 0.05

Figure 3A shows a marked (*P* < 0.05) increase in TBARS levels (an indicator of lipid peroxidation) in rat testes after administration of PDC compared with control and LCF-treated groups. Conversely, rats pretreated with LCF (200 and 300 mg/kg) and then intoxicated by PDC showed a significant (*P* < 0.05) decrease in TBARS level, particularly at the high dose as compared with PDC administrated rats.

In contrast, the GSH content, SOD, and CAT activities (Fig. 3B, C, and D) considerably (*P* < 0.05) declined in PDC-administrated rats. Supplementation of LCF at doses of 200 and 300 mg/kg before PDCadministration significantly elevated GSH content, SOD, and CAT activities in rat testes homogenate. None of the oxidative parameters of the sole LCF administered two doses (200 and 300 mg/kg) was significantly altered.

### Lactoferrin Amended Proinflammatory Cytokine Levels in PDC-Intoxicated Rats

**Fig. 4:**
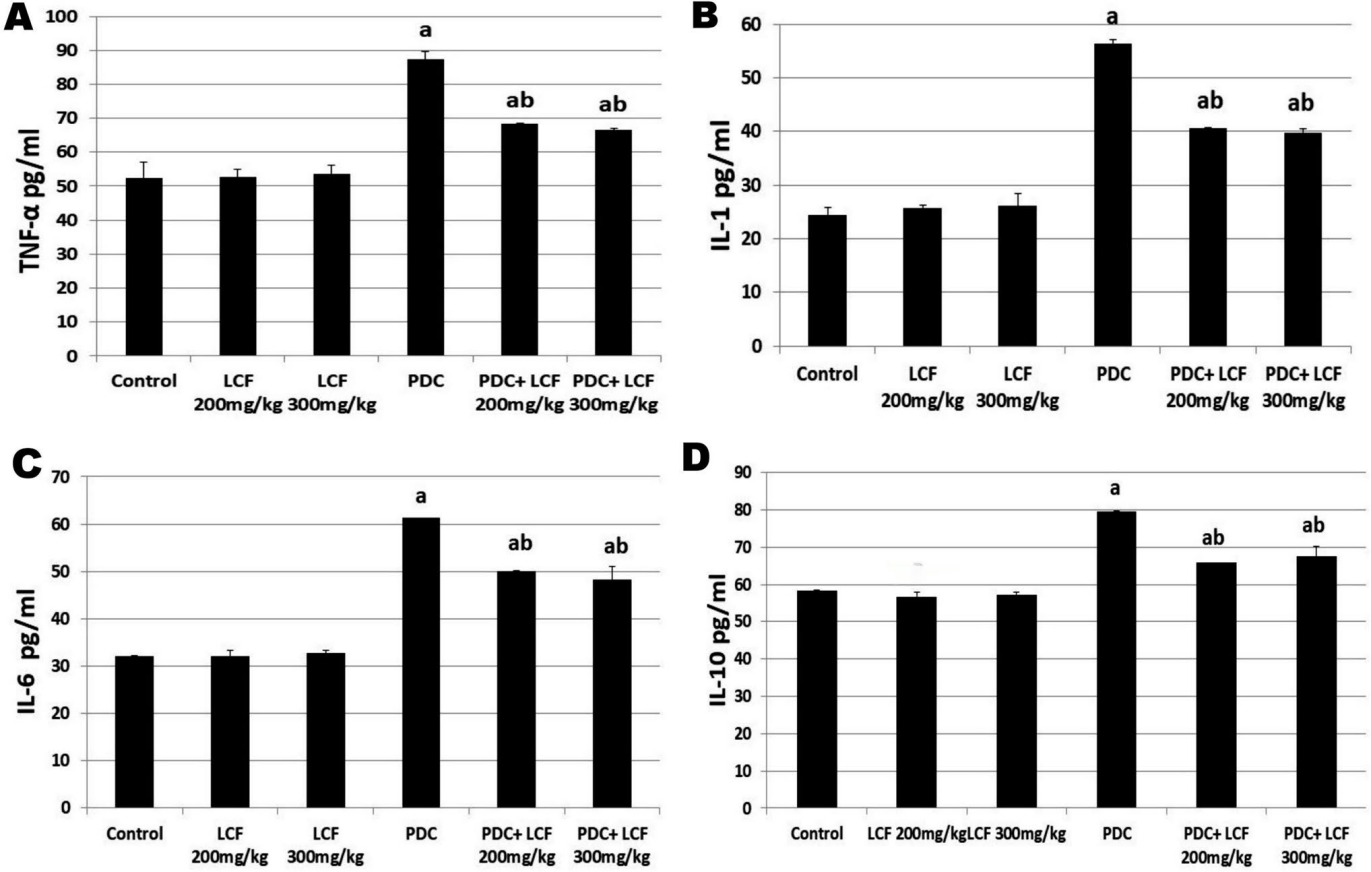
Effect of lactoferrin on the proinflammatory cytokines levels including TNF-α (**a**), IL-1 (**b**), IL-6 (**c**), and IL-10 (**d**) as well as chromium residue (**e**) in testicular tissue homogenate of PDC intoxicated rats. TNF-α as well as IL-1, IL-6, and IL-10. Values are presented as mean ± SE. ^a^ refers to statistically significant difference from the control group at *P* < 0.05. ^b^ refers to statistically significant difference from PDC administrated group at *P* < 0.05

PDC intoxication significantly (*P* < 0.05) elevated testicular levels of TNF-α as well as IL-1, IL-6, and IL-10 when compared with those levels of control rats as well as those administrated LCF alone at 200 and 300 mg/kg. Contrarily, the pre-administration of the last two doses of LCF before PDC significantly diminished PDC- induced elevation in the investigated proinflammatory cytokines compared with the PDC-intoxicated rats (Fig. 4A–D).

### LCF Improved Histopathological Alterations and Lesion Scoring in Testicular Tissue of PDC-Intoxicated Rats

**Fig. 5:**
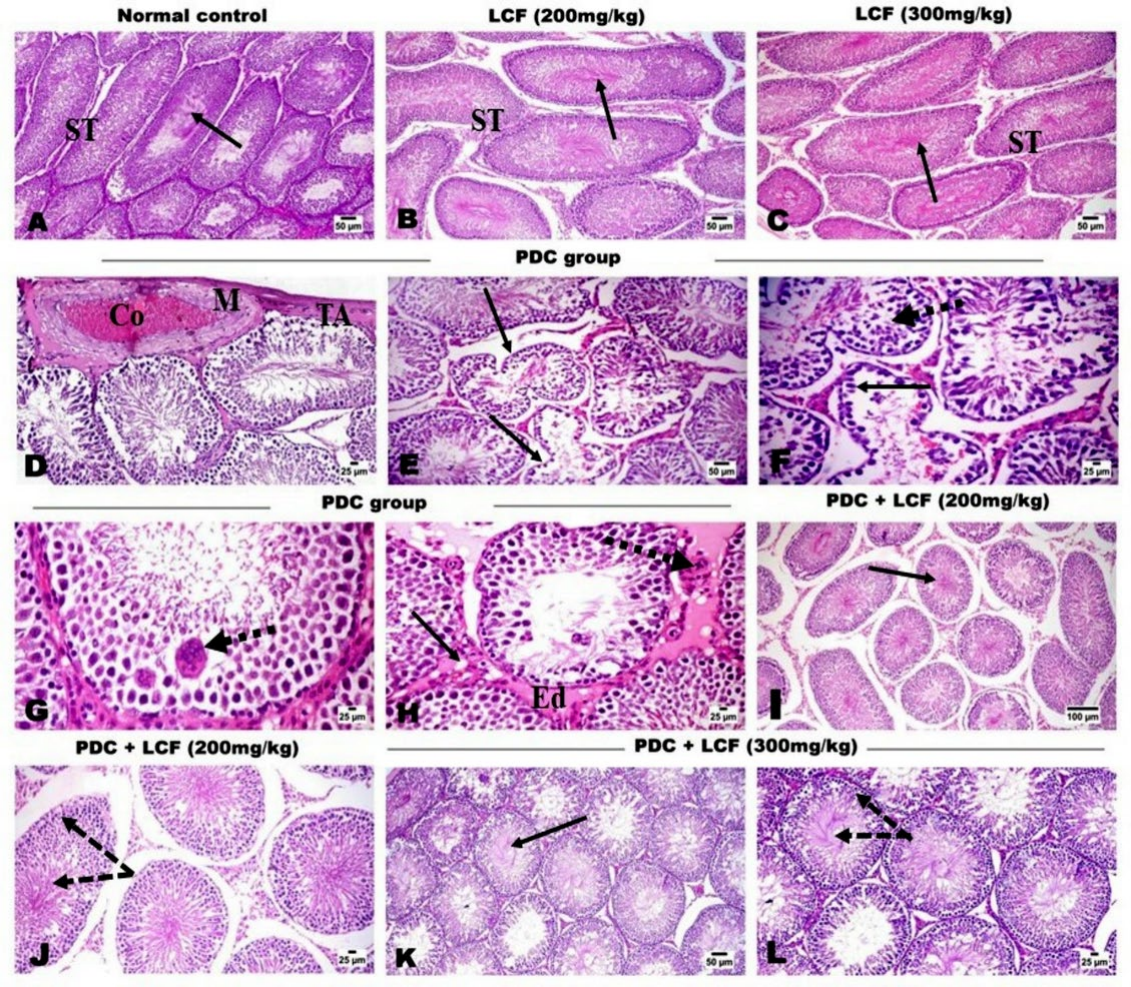
Photomicrographs of H&E-stained testicular tissue of (**a**) normal control, (**b** and **c**) LCF administrated rats (200 and 300 mg/kg) showing normal histological structure of seminiferous tubules (ST) with robust spermatogenesis and active sperms (arrow) in their lumens. (**d**–**h**) PDC intoxicated rat showing; (**d**) thickening of tunica albuginea (TA), congested (Co) and medial (M) thickening of the sub-tunical blood vessels, (**e**) irregular contour of the ST (arrow), (**f**) degenerative and necrotic changes of spermatogonial cells (dashed arrow), some tubules appeared as lined by Sertoli cells (arrow), (**g**) spermatid giant cells (arrow), and (**h**) eosinophilic edematous fluid (Ed) in the interstitial tissue with degenerated (arrow) and necrotic (dashed arrow) Leydig cells. (**i**–**l**) LCF pretreated testicular tissue at 200 (**i** and **j**) and 300 (**k** and **l**) mg/kg showing dose related preserved spermatogenesis (dashed arrow) and active sperms (arrow) in most of the ST lumens

Microscopic examination of various testicular sections of control as well as the sole LCF-administrated rats at both doses (200 and 300 mg/kg) showed the normal histological structure of seminiferous tubules (STs) with active spermatogenesis in their lumens (Fig. 5A–C). Examining the testicular tissue of rats administered PDC revealed defective spermatogenesis in the majority of seminiferous tubules with the most irregular contours. The tunica albuginea was moderately thickened, accompanied by sub-tunica congestion and edema. The sub-tunical blood vessels showed medial thickening and vacuolation (Fig. 5D). The seminiferous tubules exhibited pronounced degenerative and necrotic alterations of the spermatogoial cell layers, which frequently appeared detached from the basement membrane. Others exhibited pyknotic nuclei. Some STs appeared to be severely affected and exhibited extensive spermatogonial cell necrosis, scattered apoptosis, and prominent intraluminal spermatid giant cells (Fig. 5F), and others appeared to be lined by Sertoli cells. The interstitial spaces were expanded and showed accumulation of vacuolated to homogenous eosinophilic edematous fluid (edema), congested vessels. The Leydig cells appeared vacuolated, atrophic, or depleted (Fig. 5G). Pretreatment with LCF, either at 200 mg/kg (Fig. 5H and I) or at 300 mg/kg (Fig. 5J and K), could markedly preserve the histological picture of the testes and minimize the histological damage induced by PDC administration, particularly at the high dose. Most seminiferous tubules showed normal distribution of spermatogenic cycles with a rise in spermatozoa count.

**Fig. 6:**
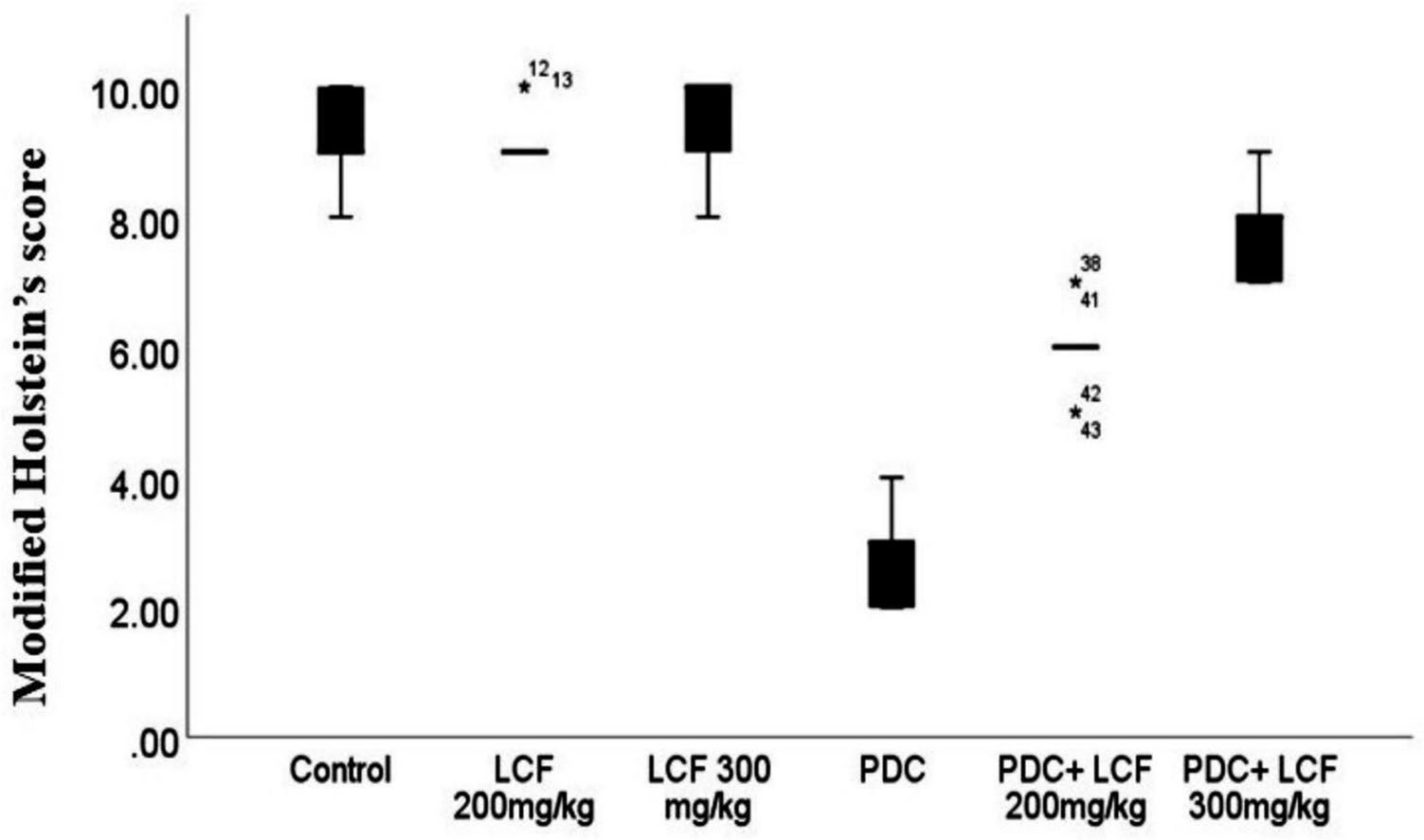
Modified Holstein’s scoring of testicular lesions in normal control and various experimental groups showing significant dose-related rise scores in LCF pretreated groups compared with that of PDC group. Data are presented as median (max-min) using Kruskal-Wallis test followed by the Mann-Whitney U test. Significant difference was considered at *P* < 0.05

In addition, PDC significantly decreased the modified Holstein score (*P* < 0.05) compared to the control group and the LCF group alone (Fig. 6). However, the pretreatment with LCF before PDC administration resulted in a significant dose-related elevation of modified Holstein’s score in comparison with that of PDC group.

By comparing the PDC-administered group to controls and the other treated groups, the morphometric analysis revealed a substantial (*P* < 0.05) decline in seminiferous tubules’ analyzed parameters (area, circumference, and mean diameter) compared to controls as well as the other treated groups. In contrast, treatment with LCF (200 and 300 mg/kg) prior to PDC administration resulted in significant (*P* < 0.05) amendement in the measured parameters (Table 3).

**Table 3:** Effect of LCF on PDC-induced changes in the morphometric parameters of seminiferous tubules (mean diameter, circumference, and area)

Groups items	Control	LCF 200 mg/kg	LCF 300 mg/kg	PDC	PDC + LCF 200 mg/kg	PDC + LCF 300 mg/kg
Diameter (µm)	238.5 ± 6.8	242.7 ± 6.1	250.4 ± 3.9	94.9 ± 3.1^a^	146.3 ± 2.4 ^ab^	191.5 ± 1.5^ab^
Circumference (× 10^−3^ m)	1.19 ± 0.03	1.19 ± 0.01	1.22 ± 0.01	1.08 ± 0.01^a^	1.14 ± 0.01^ab^	1.16 ± 0.00 ^ab^
Area (× 10^−8^m^2^)	8.74 ± 0.17	8.74 ± 0.17	9.01 ± 0.12	7.13 ± 0.11^a^	7.85 ± 0.05 ^ab^	8.1 ± 0.1^ab^

Values are presented as mean ± SE. ^a^ refers to a statistically significant difference from the control group at *P* < 0.05. ^b^ refers to a statistically significant difference from PDC administrated group at *P* < 0.05

### LCF Modulated the Immunohistochemical Expression of FasL and Nrf2 in the Testicular Tissue of PDC-Intoxicated Rats

**Fig. 7:**
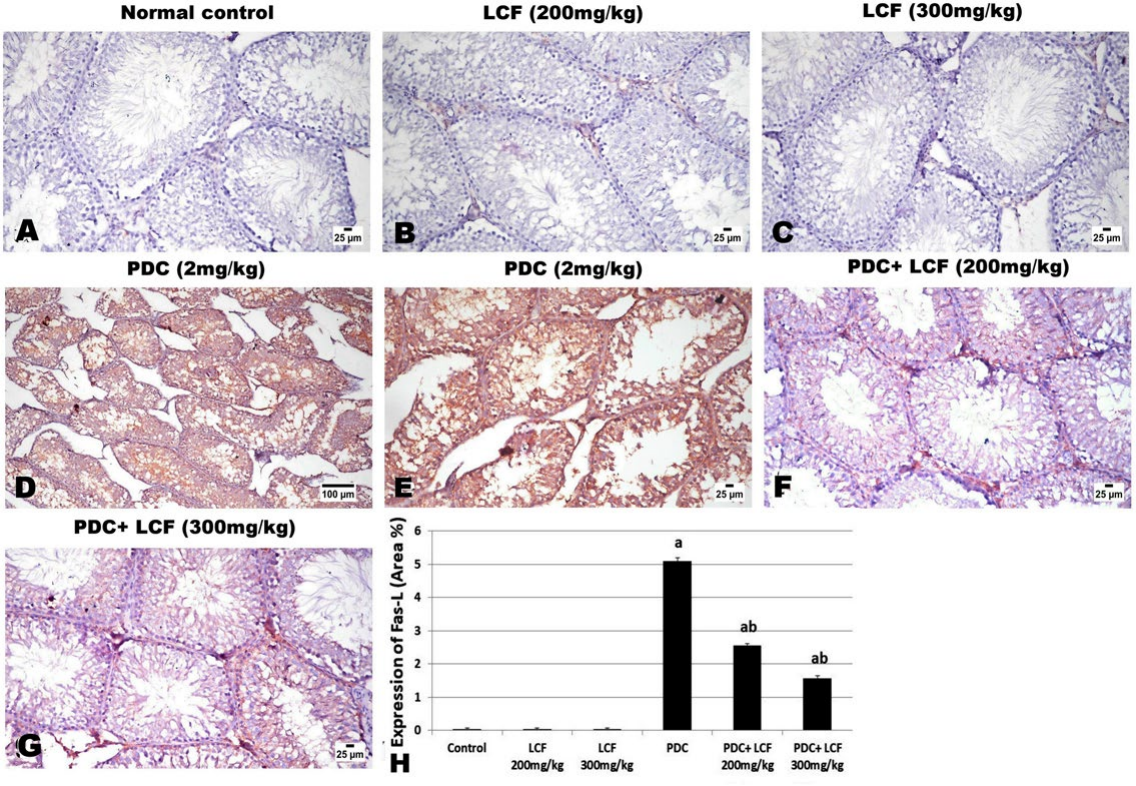
Photomicrograph of immune-stained testicular tissue for the expression of FasL showing significant increased expression in testicular tissue of PDC intoxicated rat and a dose‐related decreased expression in LCF (200 and 300 mg/kg) pretreated rats. Values are presented as mean ± SE. ^a^ refers to statistically significant difference from the control group at P < 0.05. ^b^ refers to statistically significant difference from PDC administrated group at *P* < 0.05

**Fig. 8:**
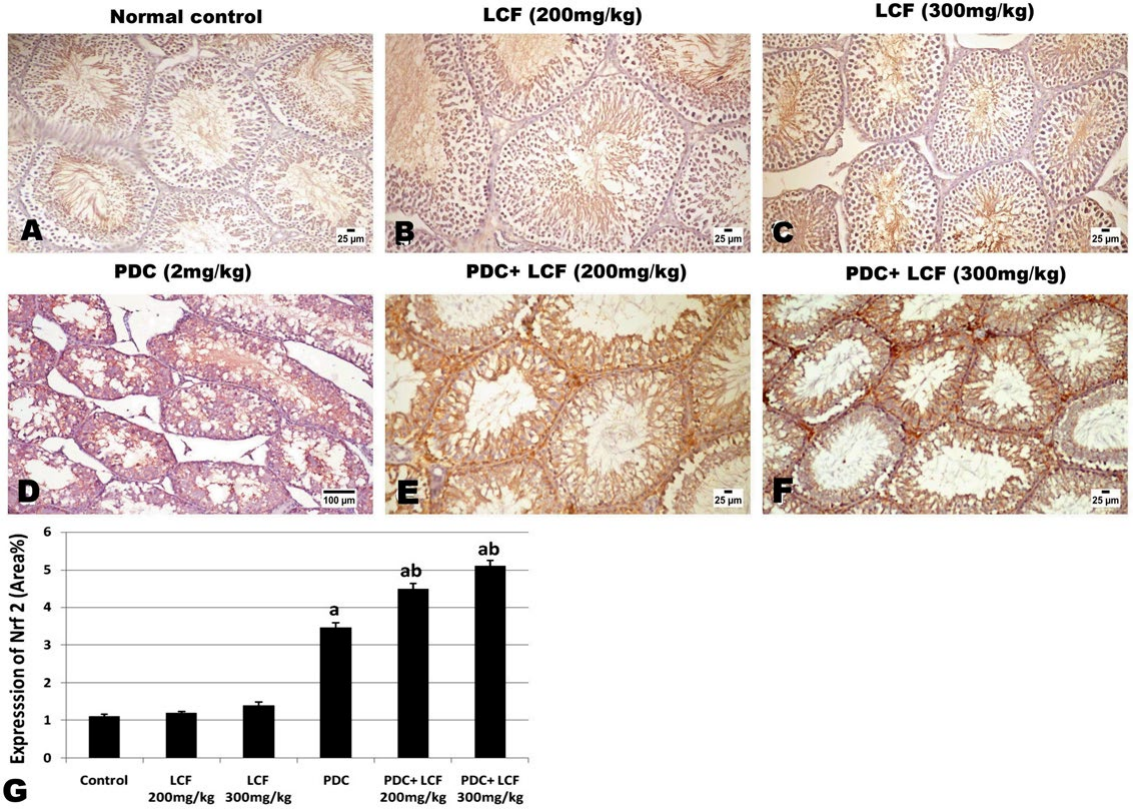
Photomicrograph of immune-stained testicular tissue for the expression of Nrf2 showing mild expression in normal control and LCF treated groups, moderate increased expression in testicular tissue of PDC intoxicated rat, and a dose‐related significant increased expression in LCF (200 and 300 mg/kg) pretreated rats. Values are presented as mean ± SE. ^a^ refers to statistically significant difference from the control group at *P* < 0.05. ^b^ refers to statistically significant difference from PDC administrated group at *P* < 0.05

Control and LCF-administered groups exhibited negative expression of Fas (Fig. 7) and mild expression of Nrf2 (Fig. 8). In the testicular tissue of the PDC-administered group, a significant (*P* < 0.05) boost in the expression of FasL and a moderate increase in the expression of Nrf-2 were observed. In contrast, a dose-dependent significant (*P* < 0.05) decrease in the expression of FasL and an increase in the expression of Nrf2 were observed in testicular tissues of LCF (200 and 300 mg/kg) pretreated groups prior to PDC administration compared to the PDC administered group, as demonstrated by image analysis of the optical density of the positive brown color of each marker expression.

## Discussion

Males’ reproductive systems are particularly sensitive to a variety of environmental factors, such as pollution, radiation, medications, and hazardous substances. Male fertility may be impacted by PDC (a source of hexavalent chromium (Cr VI) poisoning). It is well known that PDC can cause testicular toxicity, which leads to a spermatogenic arrest. PDC disrupts spermatogenesis by inducing free radical toxicity. LCF has been specified as a natural antioxidant protein that can decrease ROS formation and boost the antioxidant capacity, which could protect against the toxic effects of PDC. Therefore, the current investigation was performed to examine the potential preventive effect of LCF on testicular tissue against the toxic effect of PDC.

As far as we are aware, this research is the first to show the preventative influence of LCF against PDC-induced testicular pathology in male rats.

Male rats were injected intraperitoneally with 2 mg/kg PDC for 4 weeks, resulting in a significant decrease in body and testis weight, sperm count, motility, and viability, in addition to elevated incidence of sperm abnormalities. In addition, serum FSH levels were substantially elevated, whereas serum testosterone levels and testicular antioxidant contents considerably declined.

Moreover, PDC significantly elevated TBARS levels, markedly increased testicular cytokines and chromium contents, and induced testicular pathology, which is compatible with the outcomes of Chandra et al. [34] and Bashandy et al. [35]. Conversely, treatment with LCF (200 and 300 mg/kg/day) prior to PDC administration, protected testicular tissue from the deleterious effects of PDC. LCF could preserve testicular histological appearance, increased testicular weight, body weight, spermogram, decreased altered levels of serum hormones, decreased oxidative stress and lipid peroxidation. Additionally, LCF caused a modification in the levels of proinflammatory cytokines. There is a scarcity of data regarding the impact of LCF on chromium toxicity in the testes of rats.

Consistent with the findings of Chandra et al. [34], PDC led to a decrease in body and testicular weights. The decreased weight gain caused by PDC may be attributable to a decrease in food intake or decreased body fat and lean mass [34]. Conversely, the testicular weight reduction may be due to diminished testosterone levels since it is widely known that testosterone is responsible for developing male reproductive organs [36]. LCF prevented body weight loss caused by PDC intoxication. This result aligns with Safaeian and Zabolian [37], who found that LCF could prevent dexamethasone-induced body weight loss. Furthermore, Wakabayashi et al. [38] have mentioned the role of LCF in preventing body weight loss in mice infected with type 1 herpes simplex virus, which may be performed by immune modulation and inhibiting proinflammatory cytokines, an attribution that agrees with our results. The mended testicular weight achieved by LCF could be referred to its ability to restore serum testosterone levels.

Sperm characteristics were shown to be affected by the current administration of PDC, detected by a significant reduction in sperm viability, count, and motility, in addition to significantly increase in sperm abnormalities. This finding indicates an interference with spermatogenesis, which aligns with Sadek [39], who found that exposure to CrVI interfered with spermatogenesis, increased sperm abnormalities, and reduced sperm count. These effects may be due to the peroxidation of lipids that causes protein and DNA damage, leading to sperm degradation and infertility. In addition, Johnson and Radhakrishnan [1] attributed the observed testicular damage and inhibited spermatogenesis induced by PDC administration to its direct cytotoxic effect and the endocrine function disruption exhibited by the low level of serum testosterone. This result may explain the diminished number of epididymal sperms and the impairment of spermatogenesis, which was confirmed by our hormonal findings. Polyunsaturated fatty acids, which are abundant in spermatozoa as well as the diminished testicular antioxidant capacity may increase spermatozoa's sensitivity to free radical-induced damage, resulting in decreased sperm density and motility [40]. Additionally, ROS generated during the decline in hexavalent chromium to trivalent form may decrease the motility of spermatozoa by impacting axonemal protein phosphorylation necessary for spermatozoa movement.

LCF treatment prior to PDC administration could restore normal epididymal sperm count and seminiferous tubules’ sperm production. This result suggests that LCF has the potential to normalize spermatogenesis due to its antioxidant properties. It has been mentioned that disorders in sperm parameters can be mended by exogenous antioxidants/ROS scavengers [41]. It has been proposed that LCF aids oxidoreductive processes at cell membranes, and the process of membrane lipid peroxidation is limited to binding LCF to the cells as lactoferrin is not fully saturated. Therefore, it can scavenge free iron, a cytotoxic activator of lipid peroxidation and oxidative stress, thereby inhibiting damage caused by free radicals [42].

In males, endocrine control of reproduction begins with GnRH secretion from the hypothalamus, which stimulates the synthesis of gonadotropins (LH and FSH) from the pituitary gland. Stimulated by LH, Leydig cells release testosterone, which acts parallel with FSH to regulate Sertoli cell function [43]. The current study revealed that exposure to PDC provoked a decrease in testosterone level and an increase in FSH, which agrees with results observed by Chandra et al. [34] and Kumar [44]. As confirmed by our histopathological observations, the decreased testosterone level may be attributed to degenerative changes in Leydig cells caused by PDC. However, the rise in FSH level is probably due to lowering the production of inhibin by Sertoli cells, which negatively affects the hypothalamic-pituitary axis [45]. LCF treatment prior to PDC reversed these hormonal alternations, which may be achieved through increasing the concentrations of steroidogenesis substrates, as LCF was mentioned to cause activation of androgen synthesis and lipid metabolism [46].

Usually, reactive oxygen species (ROS) are produced as an outcome of cellular metabolism, causing damage to various structures of the cells because they are highly reactive. Through their natural antioxidants, body cells can protect themselves from ROS. Typically, there is an equilibrium between the production of ROS and their clearance by antioxidants; however, excessive production of ROS causes this equilibrium to change, known as oxidative stress [47]. Cellular antioxidants include enzymatic antioxidants like SOD and CAT and non-enzymatic antioxidants like reduced GSH. SOD converts O_2_ to H_2_O_2_, which is then destructed to water by CAT, which also binds to NADPH to prevent the oxidative inactivation of enzymes. While for antioxidative enzymes (glutathione-S-transferase and glutathione peroxidase), GSH serves as a co-substrate, it also serves as a scavenger of free radicals due to the SH group.

The oxidative stress and disturbance of the hypothalamic-pituitary–testicular axis may be involved in PDC-induced reproductive impairment. The pituitary gland is thought to be susceptible to Cr-VI toxicity [48]. Cr-VI accumulation in the hypothalamic-pituitary axis indicates that the metal may interfere with proper endocrine function. In addition, PDC treatment induces testicular oxidative stress, as indicated by elevated testicular TBARS levels. Besides a decline in testicular GSH, CAT, and SOD levels. The oxidation product, TBARS is an essential lipid peroxidation indicator because it is the primary oxidation product of peroxidized polyunsaturated fatty acids, which causes damage to biologically important molecules and tissues when elevated [49]. Testicular GSH concentration, catalase, and SOD activities significantly decreased in PDC-admmnistrated rats, indicating that the testicular defense mechanism is incapable of mitigating excessive ROS generation, which agrees with the results of Demerdash et al. [50]. PDC-induced ROS and oxidative stress, which damaged and malfunctioned spermatogenic cells. In addition, the elevated oxidative stress impairs the physiological function of Leydig cells, which play a crucial role in testosterone synthesis [43] that further explains the decreased testosterone level in the PDC group.

Generally, LCF is well known for its antioxidant activity [20, 50], as confirmed by the observed significant dose-related increase in GSH content SOD and CAT activities accompanied by a significant decrease in TBARS level compared with PDC intoxicated rats in our study.

In addition to oxidative stress, ROS are involved in a clinical disorder known as systemic inflammatory response syndrome (SIRS). Increased generation of ROS promotes the release of inflammatory cytokines, which is essential for the beginning and development of inflammation [51].

Interleukins, interferons, and TNFs (tumor necrosis factors) are all members of the cytokines’ family, which are responsible for cell death as well as inflammatory cascades. In agreement with Bashandy et al. [35], this study revealed a substantial elevation in testicular cytokine content, including interleukins (IL-1, IL-6, and IL-10) and TNF-α, in the PDC group compared to the other groups. This elevated cytokine level may result from the stimulatory effect of Cr-VI on the immune system to increases cytokine levels.

TNF-α has been shown to suppress steroidogenesis in Leydig cells by inhibiting steroidogenic enzyme transcription via activation of NF-κB [52], which helps to explain the observed decline in testosterone production in the PDC group in our results. It is well known that inflammation alters male fertility due to elevated ROS levels and oxidative stress [53]. Moreover, the relationship between oxidative stress and TNF-α is complicated since it has been proven that TNF-α raises ROS, which in turn raises raises TNF-α levels [54].

LCF is considered a first-line defense mediator that helps restore immunological homeostasis by normalizing insult-induced responses [19, 55]. The mechanism by which LCF protects testicular tissue against PDC toxicity may occur via lowering proinflammatory cytokine production among which; TNF-α and IL-6, as shown in our results.

The findings regarding chromium residues in the PDC-treated groups’ testes revealed a significant increase compared with the other groups, demonstrating the metal’s uptake after intraperitoneal injection, which matched the results of Marouani et al. [56]. The blood-testis barrier is well disrupted by CrVI with a subsequent accumulation in testicular tissue, thus resulting in pathological alterations.

In the present investigation, pathological changes such as necrosis and testicular degeneration with impaired spermatogenesis and necrosis of Leydig cells concur with those mentioned by Bashandy et al. [35]. The later histopathological changes could be attributed to an increment in the testicular TBARS level and a drop in the testicular antioxidant enzymes. Furthermore, the observed spermatid giant cell in the lumen of some seminiferous tubules may be caused by failure of cytokinesis or the perturbation of cytoplasmic bridges connecting germ cell clones that align with Aruldhas et al. [57]. The induced oxidative stress by CrVI toxicity further triggered intrinsic and extrinsic apoptotic pathways, which was confirmed by the positive immunohistochemical expression of Fas-Ligand in seminiferous tubules of PDC-intoxicated rats. Fas ligand (FasL or CD178 or CD95L) is a member of the tumor necrosis factor (TNF) family, type-II transmembrane protein. It is activated when it binds to its receptor, then further activates the caspase cascade inducing apoptosis. The reduction of hexavalent chromium to the trivalent form and production of ROS led to cell cycle arrest and apoptosis [58]. LCF has been reported to inhibit cellular apoptosis [59], as evidenced in our results by a decrease in Fas-ligand expression in the seminiferous tubules of LCF-treated groups.

Nrf2 the nuclear factor erythroid 2-related factor 2) has been hypothesized to be an important regulator of antioxidant defense to defend against insult-induced organ damage. Nrf2 regulates the pathological and physiological impacts of oxidant exposure by stimulating the expression of a variety of antioxidant response element–dependent genes [60]. Immunohistochemical analysis of the present work revealed relatively increased expression of Nrf2 in the testicular tissue of the PDC intoxicated group. These results align with that of Shaw et al. [61], who illustrated that Cr-VI exposure raises Nrf2 expression, which is thought to be a compensatory mechanism for oxidative stress to counterbalance chromium’s harmful effects. This result might be explained by the fact that Cr causes the production of ROS and raises oxidative stress, thus increasing Nrf2 as one of the transcription factors.

LCF’s metal ions-binding capacity protects against pathogenic conditions related to iron-catalyzed ROS [62]. LCF is an important specialized iron scavenger, and its capacity to bind ferrous and ferric ions is most likely linked to its antioxidant action. Moreover, LCF may reduce the generation of hydroxyl radicals via the Fenton reaction, which is a major source of ROS [55]. In the current work, LCF treatment to PDC intoxicated rats improved the histological appearance of their testes, suppressing the harmful effects of PDC, probably due to its antioxidant activity. Our results regarding Nrf2 immunoexpression in LCF treated groups revealed significantly higher expression in testicular tissue than in the PDC-intoxicated group. It was reported that LCF increases Nrf2 expression by activating the Keap1/Nrf2 signaling pathway [63]. Generally, the antioxidant defense system of cells is controlled by a complex network of transcription factors that coordinate the expression of the genes that code for antioxidant enzymes. Nrf2 is thought to be the master regulator of antioxidant genes such as catalase and SOD [60], which explains our currently mentioned result of increased testicular antioxidant enzyme system in LCF pretreated groups.

## Conclusion

The current study clearly highlights the role of LCF as a protective agent against the oxidative damage of chromium on the testicles. The LCF exerts its sparing effect by sequestering the intermediate reactive species and hence curtailing the developing PDC-induced oxidative stress and enhancing an important regulator of antioxidant defense (Nrf2). Stopping the cascade of immunologic destruction of the testicular tissue is achieved via reducing the production of inflammatory cytokines, especially IL-1, IL6, IL-10, and TNF-α. LCF successfully filled each of these actions, establishing its role as a preferable choice for guarding testicular tissue against chromium toxicity.

## Data Availability

The data used to support the findings of the current study are available from the corresponding author upon reasonable request.

## Abbreviations

LCF: Lactoferrin
PDC: Potassium dichromate
Cr: Chromium
Cr-VI: Chromium hexavalent
Cr-III: Chromium trivalent
CAT: Catalase
SOD: Superoxide dismutase
GSH: Glutathione
TBARS: Thiobarbituric acid-reactive substances
FSH: Follicle stimulating hormon
IL-1: Interlukine-1
IL-6: Interlukine-6
IL-10: Interlukine-10
TNF-α: Tumor necrosis factor-α
FasL: Fas ligand (a homotrimeric type II transmembrane protein that belongs to the tumor necrosis factor (TNF) family
Nrf2: Nuclear factor erythroid 2-related factor 2
DAB: Diaminobenzidine
ROS: Reactive oxygen species
GnRH: Gonadotropin-releasing hormone
LH: Luteinizing hormone
NADPH: Nicotinamide adenine dinucleotide phosphate
SIRS: Systemic inflammatory response syndrome
NF-κB: Nuclear factor kappa B
Keap1: Kelch-like ECH-associated protein 1
